# RNA-RNA interaction prediction using genetic algorithm

**DOI:** 10.1186/1748-7188-9-17

**Published:** 2014-06-29

**Authors:** Soheila Montaseri, Fatemeh Zare-Mirakabad, Nasrollah Moghadam-Charkari

**Affiliations:** 1 Department of Mathematics, Statistics and Computer Sciences, University of Tehran, Tehran, Iran; 2 Faculty of Mathematics & Computer Science, Amirkabir University of Technology, Tehran, Iran; 3 School of Biological Science, Institute for Research in Fundamental Sciences (IPM), P.O. Box: 19395- 5746, Tehran, Iran; 4 Faculty of Electrical & Computer Engineering, Tarbiat Modares University, Tehran, Iran

**Keywords:** RNA secondary structure, Minimum free energy, Fitness function

## Abstract

**Background:**

RNA-RNA interaction plays an important role in the regulation of gene expression and cell development. In this process, an RNA molecule prohibits the translation of another RNA molecule by establishing stable interactions with it. In the RNA-RNA interaction prediction problem, two RNA sequences are given as inputs and the goal is to find the optimal secondary structure of two RNAs and between them. Some different algorithms have been proposed to predict RNA-RNA interaction structure. However, most of them suffer from high computational time.

**Results:**

In this paper, we introduce a novel genetic algorithm called GRNAs to predict the RNA-RNA interaction. The proposed algorithm is performed on some standard datasets with appropriate accuracy and lower time complexity in comparison to the other state-of-the-art algorithms. In the proposed algorithm, each individual is a secondary structure of two interacting RNAs. The minimum free energy is considered as a fitness function for each individual. In each generation, the algorithm is converged to find the optimal secondary structure (minimum free energy structure) of two interacting RNAs by using crossover and mutation operations.

**Conclusions:**

This algorithm is properly employed for joint secondary structure prediction. The results achieved on a set of known interacting RNA pairs are compared with the other related algorithms and the effectiveness and validity of the proposed algorithm have been demonstrated. It has been shown that time complexity of the algorithm in each iteration is as efficient as the other approaches.

## Background

Major successes have been achieved in the treatment of some cancers, including colon, breast and pancreatic by suppressing the gene expression involved in the development of these diseases using RNA-RNA interaction. The interaction between two RNAs is known as the newest and the most efficient method for gene silencing. It has been shown that the small interfering RNAs (siRNAs) can be used for silencing their target mRNAs [1]. Furthermore, small RNAs (sRNAs) play an important role in the regulation of gene expression. They usually bind to their target mRNAs to prevent their translation [2].

In RNA-RNA Interaction Prediction (RRIP) problem, two RNA sequences are given as inputs and the goal is to find the minimum free energy Secondary Structure of Interacting RNAs (SSIR). To tackle this problem, some algorithms have been proposed by research groups. Andronescu et al. [3] proposed a method based on dynamic programming in which two RNA sequences are concatenated as a single sequence and its secondary structure is calculated [3]. Another approach calculates the partition function of a secondary structure complex of multiple interacting RNAs [4]. This method rigorously extends those models of secondary structure to the multi-stranded case. The tools such as RNAhybrid [5], UNAFold [6] and RNAduplex from *ViennaRNA package*[7] reduce computational time complexity by ignoring all the internal base pairings in both RNAs. RNAup [8,9] extends the standard partition function approach to RNA secondary structures and employs the single (unpaired) regions on each RNA to find the interaction between them. RNAplex [10,11] finds the possible hybridization sites for RNA in the large RNA databases based on a slight simplification of the energy model. In this model, the loop energy is assumed to be a function of the loop size.

Recently, a novel algorithm based on the multiple context free grammars was introduced in [12]. Accordingly, two real values called transition and emission probabilities are specified for each rule of the grammar. Then, a derivation tree is constructed for the grammar based on the rules with high probability.

In heuristic based approaches, inRNAs [13] firstly predicts the loop regions in the native structure of each sequence, and then finds the optimal non-conflicting interaction between two RNAs. IntaRNA [14] combines the accessibility of target sites as well as the existence of a user-definable seed to find RNA-RNA interaction. Minimizing the joint free energy between two RNA molecules under a number of energy models with growing complexity was introduced in [2]. Another interesting heuristic approach for this problem was presented in [15]. This algorithm employs some dot matrices representation of all possible base pairs for finding the secondary structure of each RNA and between the two RNAs.

An approximation algorithm was presented in [1], where an RNA-RNA interaction graph is created in which every edge represents a possible bond in or between two RNAs. A set of edges is found to maximize the number of bonds. A statistical sampling algorithm was introduced in [16] based on some modifications to the grammars. It calculates the interaction probabilities for any given single region on RNA. RactIP [17] predicts RNA-RNA interaction using integer programming. Accordingly, it uses the approximate information of the internal and external base pairing probabilities of joint structures as an objective function of integer programming. PETcofold [18] employs covariance information in the internal and external base pairs to predict SSIR of two multiple alignments of RNA sequences. InteRNA [19] reduces the time and space complexity of RRIP problem described by Alkan et al. [2] using dynamic programming sparsification.

One of the pitfalls of the most existing algorithms is their high computational time to predict RNA-RNA interaction, while a number of them have not been performed on some RNA pairs to predict binding sites between two single regions of RNAs. Alkan et al. [2] proved that RNA-RNA interaction prediction is an NP-complete problem.

In this paper, we propose a new genetic algorithm called GRNAs as an appropriate solution for the RRIP problem. This algorithm can be performed on some standard RNA pairs with high accuracy. In this method, at first, all possible stems in each RNA as well as all possible hybrid regions between two RNAs are extracted from a dot matrix. The initial population consists of some individuals, where each of them is an SSIR obtained from some randomly extracted stems and hybrid regions of the dot matrix. The minimum free energy is computed for each individual as a fitness value. For each generation, some individuals are selected to mate based on their fitness values and form a new population. Then, mutation operation is done on a few individuals. The population generation terminates when the free energy of an individual is minimum enough. Finally, one of the best individuals is selected as an optimal SSIR. The algorithm is conducted on some real datasets and compared with some other algorithms to investigate efficiency and validity of the proposed method. The time and space complexity of the proposed method in each iteration is 0(*l*^2^ + |*P*|), where *l* and |*P*| indicate the sum of the length of two RNAs and the length of an individual, respectively. The results show that the accuracy of the algorithm is as efficient as the other related methods.

The rest of this paper is organized as follows. In Section 2, some definitions and notations are described. In Section 3, a genetic algorithm called GRNAs is presented to predict RNA-RNA interaction. The results and conclusion are discussed in Sections 4 and 5, respectively.

## Definitions and notations

An RNA molecule is composed of a long, usually single-stranded chain of nucleotide units; adenine (*A*), cytosine (*C*), guanine (*G*) and uracil (*U*). Thus, *R* = *r*_1_*r*_2_ … *r*_*n*_ in 5 ' - 3 ' direction is an RNA sequence, where |*R*| = *n* and *r*_*i*_ ∈ {*A*, *C*, *G*, *U*} (1 ≤ *i* ≤ *n*). The RNA structure is formed by the creation of hydrogen bonds between Watson-Crick complementary bases (*A* - *U* and *C* - *G*) and a Wobble base pair (*G* - *U*).

In an RNA secondary structure, each base interacts with at most one other base, and no base pairs cross each other. Two bases *r*_*i*_ and *r*_*j*_ (1 ≤ *i* < *j* ≤ *n*) of the base pair (*r*_*i*_, *r*_*j*_) are represented by ' (' and ') ', respectively and each unpaired base is declared by '. '. A stem consists of subsequent base pairs and a loop is one sequence of consecutive unpaired bases.

A secondary structure of two interacting RNAs, *R*_1_ and *R*_2_, contains the set of stems in each RNA and the hybrid regions between two RNAs as well as loops. Each hybrid region consists of subsequent hybrid base pairs between two RNAs. Two bases *r*_*i*_ ∈ *R*_1_ and *r*_*j*_ ∈ *R*_2_ of the hybrid base pair (*r*_*i*_, *r*_*j*_) are represented by ' [' and '] ', respectively.

Example. Let *R*_1_ = CGGUUUGAGGUCCG and *R*_2_ = ACUACCGAAAAGUU be two RNA sequences. The SSIR of the two RNAs is shown as follows;

$$\begin{array}{cc} 5'-\text{CGGUUUGAGGUCCG}-3' & 5'-\text{ACUACCGAAAAGUU}-3'\\ \left(\;(\;(\;[\;[\;[\;..\;[\;[\;[\;)\;)\;\right) & \left(\;\left(\;\left(\;]\;]\;]\;..\;]\;]\;]\;\right)\;\right)\;\right) \end{array}$$

In this example, each RNA has one stem. In the left hand RNA, one stem is found by the production of bonding between *CGG* and the reverse *CCG* (*GCC*). There are two hybrid regions between the sequences *R*_1_ and *R*_2_. The first one is produced by binding between *UUU* and the reverse *AAA* (*AAA*). The second one is generated by binding between *GGU* and the reverse *ACC* (*CCA*).

## A new genetic algorithm for RNA-RNA interaction prediction

Genetic algorithm is an optimization method based on evolutionary biology that is widely used to solve search and optimization problems [20-22]. In this section, a new genetic algorithm, GRNAs, is presented to predict RNA-RNA interaction. In the following, initial population, fitness function, crossover and mutation operations are introduced.

### Initial population

In the proposed algorithm, two RNA sequences *R* ' = *r* ' _1_*r* ' _2_ … *r* ' _*n*_ (|*R* ' | = *n*) and *R* " = *r* " _1_*r* " _2_ … *r* " _*m*_ (|*R* " | = *m*) are given as inputs. The two RNAs *R* ' and *R* " are converted to the sequence *R* = *r*_1_*r*_2_ … *r*_*l*_ as follows;

$$r_{i}=\left\{\begin{array}{cc} r{'}_{i} & 1\leq i\leq n,\\ r{"}_{i-n-1} & n+2\leq i\leq l\\ N & i=n+1, \end{array}\right),$$

where *N* is an arbitrary character to distinguish between two sequences and *l* = *m* + *n* + 1.

A dot matrix $M_{l\times l}^{R}$ is made, where the axes in the dot matrix correspond to the two sequences *R* and reverse *R*,as follows;

$$M^{R}\left[i,j\right]=\left\{\begin{array}{cc} 1 & \mathrm{if}\;\left(r_{i},r_{l-j+1}\right)\in \left\{\left(A,U\right),\left(U,A\right),\left(C,G\right),\left(G,C\right),\left(U,G\right),\left(G,U\right)\right\},\\ 0 & \mathrm{else}, \end{array}\right)$$

where *r*_*i*_ and *r*_*l*-*j*+1_ (1 ≤ *i*, *j* ≤ *l*) are the *i*-th and *l*-*j*+1-th nucleotides in the sequence *R* = *r*_1_*r*_2_ … *r*_*l*_, respectively. Each right-skewed consecutive value of 1’s which is parallel to the main diagonal in the dot matrix is selected as a sub-diagonal. Each sub-diagonal shows a possible stem in each RNA or hybrid region between two RNAs. Set *D*^*R*^ shows all sub-diagonals in the dot matrix as follows;

$$D^{R}=\left\{<i,j,t>\begin{array}{cc} | & 1\leq i\leq l\&1\leq j\leq l\&1\leq t\leq l-1 \end{array}\right\},$$

where *i* and *j* indicate the start position of the row and the column of a sub-diagonal with *t*+1 consecutive 1’s, respectively. Hence, each <*i*,*j*,*t*> is a set of consecutive base pairs as follows;

$$<i,j,t>=\left\{\left(r_{i},r_{l-j+1}\right),\cdots ,\left(r_{i+t},r_{l-j-t+1}\right)\right\},$$

According to the prior knowledge, we know that Watson-Crick base pairs occur more than Wobble in RNA structures. In this regard, we compute the percent of *G-U* pairs on our dataset approximated 14%. So, *G-U* pairs are removed from the sub-diagonals including more than 14% *G-U* pairs. For each *d*_1_ = < *i*_1_, *j*_1_, *t*_1_ > ∈ *D*^*R*^ and *d*_2_ = < *i*_2_, *j*_2_, *t*_2_ > ∈ *D*^*R*^, *d*_1_ ∝ *d*_2_ is defined as;

$$d_{1}\propto d_{2}=\left\{\left(r_{i},r_{j}\right)\in d_{1}|\exists \left(r_{k},r_{g}\right)\in d_{2},i=k\vee i=g\vee j=k\vee j=g\right\},$$

where *d*_1_ ∝ *d*_2_ represents all base pairs in *d*_1_ overlapping with *d*_2_.

For each individual *P*, a |*D*^*R*^| -tuple is randomly made as;

$$I=<x_{1},x_{2},\cdots ,x_{\left|D^{R}\right|}>,\cdot x_{k}\in \left\{0,1\right\},$$

where *x*_*k*_ (1 ≤ *k* ≤ |*D*^*R*^|) indicates the *k*-th sub-diagonal in *I*. In other words, individual P contains those sub-diagonals that their related *x* in *I* is equal to 1. Here, *x*_*k*_ = 1 means sub-diagonal *d*_*k*_ ∈ *P*, while *x*_*k*_ = 0 points to *d*_*k*_ ∉ *P*. Then, the individual *P* is constructed as follows;

(1) $$P=\left\{C_{k}|\;x_{k}=1,C_{k}\neq \phi \right\},$$

where the set *C*_*k*_ (modified *k*-th sub-diagonal) is obtained as follows;

$$C_{k}=\left\{\left(r_{i},r_{j}\right)\in d_{k}\right\}-\begin{array}{cc} \cup_{\begin{array}{c} {}_{1\leq t\leq k-1}\\ C_{t}\in P \end{array}} & d_{k}\propto C_{t} \end{array}.$$

Here, *C*_*k*_ ∈ *P* is a set of base pairs in *d*_*k*_ without any common base pairs in the previous sub-diagonals, *d*_*t*_(1 ≤ *t* < *k*), of individual *P*. Finally, *C*_*k*_ is modified by removing the lonely base pairs from it as follows;

$$C_{k}=C_{k}-\left\{\left(r_{i},r_{j}\right)\in C_{k}|\;\left(r_{i+1},r_{j-1}\right)\notin C_{k}\;\&\left(r_{i-1},r_{j+1}\right)\notin C_{k}\right\}.$$

Notice that a set of the produced individuals creates an initial population.

### Fitness function

For each individual *P*, let *S* and *H* represent two RNAs secondary structures and binding sites between the two RNAs, respectively. Therefore, the fitness function is defined as follows;

$$\mathrm{Fitness}\left(P\right)=\mathrm{MFE}\left(S\right)+\;\mathrm{MFE}\left(H\right),$$

where for *C* ∈ {*S*, *H*}, *MFE*(*C*) denotes the minimum free energy of structure *C*. We apply RNAeval.exe [7] to compute minimum free energies of secondary structures and binding sites separately.

### Crossover

Crossover operation is performed between the individuals with the rate of 0.9. The good and mediocre individuals are transferred to the next population. The remaining individuals are consecutively selected for crossover operation.

Let *P*_1_ and *P*_2_ are selected as parents. For each individual *P*_*i*_, 1 ≤ *i* ≤ 2, a |*D*^*R*^| -tuple is defined as follows:

$$I_{i}=<x_{i1},x_{i2},\cdots ,x_{i|D^{R}|}>,\;x_{\mathrm{ij}}=\left\{\begin{array}{cc} 1 & \mathrm{if}\;C_{j}\in P_{i},\\ 0 & \mathrm{else}.\; \end{array}\right)$$

In this procedure, a random position *k,* 1 ≤ *k* ≤ |*D*^*R*^|, is selected and *I*_1_ and *I*_2_ are crossed. Then, *I* ' _1_ and *I* ' _2_ are formed as follows:

$$\begin{array}{l} I{'}_{1}=<x_{11},x_{12},\cdots ,x_{1k},x_{2\left(k+1\right)},\cdots ,x_{2|D^{R}|}>,\\ I{'}_{2}=<x_{21},x_{22},\cdots ,x_{2k},x_{1\left(k+1\right)},\cdots ,x_{1|D^{R}|}>. \end{array}$$

In the following, two new individuals *P* ' _1_ and *P* ' _2_ are generated from *I* ' _1_ and *I* ' _2_ similar to the described method in the initial population (refers to the Equation 1), respectively.

### Mutation

Mutation operation is done with the rate of 0.1 on a few randomly selected individuals in each generation. Assume that *P is* an individual selected for mutation. For the individual *P*, a |*D*^*R*^| -tuple is obtained by:

$$I=<x_{1},x_{2},\cdots ,x_{\left|D^{R}\right|}>,\;x_{i}=\left\{\begin{array}{cc} 1 & \;\mathrm{if}\;C_{i}\in P,\\ 0 & \;\mathrm{else}.\; \end{array}\right)$$

An item *x*_*j*_, where *x*_*j*_ = 1 is randomly selected and replaced to 0. Then, another *x*_*k*_ (*x*_*k*_*=* 0) is replaced to 1. The new individual, *P*, is obtained from the changed *I* based on the proposed method in initial population (refers to the Equation 1). Finally, if *C*_*k*_ ∉ *P* (all the base pairs of *d*_*k*_ have overlapping with the existence sub-diagonals in *P*), the other *x*_*i*_ (*x*_*i*_ = 0) is selected to replace with 1. This process continues until *C*_*k*_ ∈ *P* or the defined number of generations is reached. When mutation is performed on a number of individuals, they will be increasingly sorted based on their fitness values.

### Termination of the GRNAs algorithm

The GRNAs algorithm terminates when the best individual in definite generations will not be changed or the defined number of generations be reached. After the termination of the algorithm, one of the best individuals is selected as the best folding of two RNAs and the best interaction between the two RNAs.

### Time and space complexity

We have obtained the time and space complexity of GRNAs in each iteration. Making the dot matrix needs the complexity of *O*(*l*^2^) where *l* exposes sum of the length of the two RNAs. Let *h* and |*P*| be the number of individuals and the length of an individual *P*. The time complexity of creating the initial population is *O*(*h*. |*P*| ^2^). We set *h* = 40 and |*P*| = max{|*R* ' |, |*R* " |}, so *h* can be ignored. Sorting individuals based on their fitness values requires *O*(|*P*|. *h*. log *h*). Crossover and mutation operations take *O*(|*P*| ^2^) and *O*(|*P*|), respectively. Thus, the time complexity in each iteration in the proposed algorithm is *O*(*h*. |*P*|(|*P*| + log *h*)). The maximum number of iteration is at most *I* = 20. Therefore, the time complexity of the algorithm is *O*(*I. h*. |*P*|(|*P*| + *log h*) + *l*^2^) that is simplified with *O*(*l*^2^ + |*P*|).

On the other hand, for storing the *h* individuals of length |*P*| we need *O*(*h*. |*P*|) space complexity. Furthermore, the population in the algorithm uses both dot matrix and an array of sub-diagonals. Hence, the storage complexity of these two types is *O*(*l*^2^), where *l* denotes sum of the length of the two RNAs. Thus the total space complexity of GRNAs is *O*(*h*|*P*| + *l*^2^) which is simplified with *O*(*l*^2^ + |*P*|).

## Results and discussion

The GRNAs has been performed on a machine with two-Core Intel(R) Duo processor T6670 2.20 GHz and 4 GB RAM to predict the interaction structure between two RNAs. The proposed genetic algorithm is performed on two well-known datasets of RNA-RNA interactions. The first set contains: R1inv-R2inv, Tar-Tar*, DIS-DIS, CopA-CopT and IncRNA_54_-RepZ in the *Escherichia coli* bacteria [12]. The joint secondary structures of this dataset include kissing hairpins. We evaluate the performance of joint secondary structure prediction of this dataset.

Also, this algorithm is carried on the second set of datasets with their binding sites including some RNA pairs called: DsrA-Rpos, GcvB-argT, GcvB-dppA, GcvB-gltI, GcvB-livK, GcvB-livJ, GcvB-oppA, GcvB-STM4351, IstR-tisAB, MicA-ompA, MicA-lamB, MicC-ompC, MicF-ompF, OxyS-fhlA, RyhB-sdhD, RyhB-sodB, SgrS-ptsG and Spot42-galK [14]. This dataset is used to appraise the performance of RNA-RNA interaction prediction in binding sites.

To evaluate the prediction accuracy of the GRNAs, F-measure (*F*) and Matthews Correlation Coefficient (*MCC*) [18] are calculated using sensitivity (*Sn*) and positive predictive value (*PPV*). Assume that the number of correctly predicted base pairs, the number of false predicted base pairs and the number of unpredicted base pairs are indicated by *TP, FP* and *FN*, respectively. So, *Sn*, *PPV*, *F*, and *MCC* are defined as follows:

$$\mathrm{Sn}=\;\mathrm{TP}/\left(\mathrm{TP}+\mathrm{FN}\right),\;\text{PPV}=\;\mathrm{TP}/\left(\mathrm{TP}+\mathrm{FP}\right),$$

$$F=\left(2\times \mathrm{Sn}\times \text{PPV}\right)/\left(\mathrm{Sn}+\text{PPV}\right),$$

$$\text{MCC}=\sqrt{\mathrm{Sn}\times \text{PPV}.}$$

Table 1 shows the results of joint secondary structure prediction of our algorithm, GRNAs, in Matthews Correlation Coefficient [18]. It is also compared to the state-of-the-art methods such as PETcofold [18], the sparsified version of inteRNA [19], Pairfold [3], RactIP[17] and RNAcofold [7]. The *MCC* evaluates the joint structure, i.e. both the binding sites between the two RNAs and the secondary structure of each single RNA. In two pairs MicA-ompA and OxyS-fhlA, PETcofold has the best *MCC* value and in other two pairs RyhB-uof-fur and RyhB-sodB, GRNAs has the highest *MCC* value.

**Table 1:** **The results of joint secondary structure prediction of GRNAs in *MCC *** **in comparison to the PETcofold and other joint structure prediction methods such as RNAcofold, inteRNA, Pairfold and RactIP**

**RNA-RNA pairs**	**GRNAs**	**PETcofold**	**RNAcofold**	**inteRNA**	**Pairfold**	**RactIP**
MicA-ompA	67	87	80	49	86	57
OxyS-fhlA	60	80	61	64	61	48
RyhB-uof-fur	56	13	21	12	21	19
RyhB-sodB	74	67	65	70	65	65
Average	64	62	57	49	58	47

We also compared GRNAs with four state-of-the-art methods: inRNAs, IntaRNA, RNAup and RactIP. Table 2 shows the results of prediction in binding sites in sensitivity and positive predictive values on the datasets [12,14] using the proposed approach and mentioned methods. Here, only external base pairs are considered to measure accuracy. According to the second half of Table 2, the average positive predictive value on datasets [12,14] is 96.03%. This table shows that our method is comparable with the existing methods. Table 3 indicates the accuracy of GRNAs in F-measure with considering binding sites. In GRNAs, the average F-measure is 89%. The results shown in Tables 2 and 3 indicate that GRNAs works as efficient as the other methods in average sensitivity, positive predictive value and F-measure.

**Table 2:** **The results of binding sites prediction of GRNAs in sensitivity and positive predictive value on the datasets**[12,14]**in comparison to inRNAs, IntaRNA, RNAup and RactIP**

**Sensitivity**	**PPV**									
**RNA-RNA pairs**	**GRNAs**	**inRNAs**	**IntaRNA**	**RNAup**	**RactIP**	**GRNAs**	**inRNAs**	**IntaRNA**	**RNAup**	**RactIP**
Tar-Tar*	100	100	100	100	81.5	100	83.3	83.3	83.3	57.9
R1inv-R2inv	100	100	100	100	100	100	77.8	100	77.8	100
DIS-DIS	100	100	100	100	75	100	100	100	100	78.3
CopA-CopT	89.47	88.9	100	55.6	100	80.95	82.8	39.1	65.2	100
IncRNA_54_-RepZ	71.44	100	73.8	75	100	100	88.9	85	85.7	83.3
DsrA-Rpos	73.08	80.8	80.8	80.8	65.4	100	77.8	77.8	77.8	73.9
GcvB-argT	87.5	95	95	90	95	100	86.4	95	94.7	100
GcvB-dppA	94.12	100	100	100	94.1	100	85	58.6	45.9	59.3
GcvB-gltI	91.67	75	0	0	100	95.65	50	0	0	100
GcvB-livK	70.83	54	54.2	54.2	95.5	89.47	57	56.5	56.5	95.5
GcvB-livJ	95.45	63.4	95.5	95.5	95.8	95.45	82.4	95.5	95.5	95.8
GcvB-oppA	90.91	100	100	100	100	100	73.3	95.7	95.7	100
GcvB-STM4351	72	76	76	88	88	100	100	90.5	95.7	100
IstR-tisAB	69.44	72.2	87.9	66.7	77.8	100	100	96	100	100
MicA-ompA	93.75	100	100	100	87.5	100	100	100	100	87.5
MicA-lamB	82.61	100	100	82.6	56.5	90.48	100	82.1	70.4	86.7
MicC-ompC	90.91	100	100	72.7	72.7	100	100	53.7	41	88.9
MicF-ompF	100	96	96	80	83.3	100	96	96	95.2	76.9
OxyS-fhlA	80	81.3	50	37.5	56.3	86.96	100	100	100	81.8
RyhB-sdhD	58.82	61.8	58.8	79.4	82.4	95.24	95.5	100	79.4	82.4
RyhB-sodB	100	100	100	100	100	100	100	81.8	90	39.1
SgrS-ptsG	60.87	56.6	73.9	73.9	83.9	87.5	76.5	100	100	100
Spot42-galK	61.36	43.2	40.9	52.3	68.2	87.1	76	64.3	52.3	69.8
Average	84.1	84.5	81.9	77.6	84.7	96.03	86.5	80.5	78.4	85.1

**Table 3:** **The results of binding sites prediction of GRNAs in F-measure on the datasets**[12,14]**in comparison to inRNAs, IntaRNA, RNAup and RactIP**

**RNA-RNA pairs**	**GRNAs**	**inRNAs**	**IntaRNA**	**RNAup**	**RactIP**
Tar-Tar*	100	90.9	90.9	90.9	67.7
R1inv-R2inv	100	87.5	100	87.5	100
DIS-DIS	100	100	100	100	76.6
CopA-CopT	85	85.7	56.2	60	100
IncRNA_54_-RepZ	82.92	94.1	79	80	90.9
DsrA-Rpos	84.44	79.3	79.3	79.3	69.4
GcvB-argT	93.33	90.5	95	92.3	97.4
GcvB-dppA	96.97	91.9	73.9	62.9	72.2
GcvB-gltI	93.62	60	0	0	100
GcvB-livK	76.07	55.5	55.3	55.3	95.5
GcvB-livJ	95.45	71.7	95.5	95.5	95.8
GcvB-oppA	95.24	84.6	97.8	97.8	100
GcvB-STM4351	83.72	86.4	82.6	91.7	93.6
IstR-tisAB	81.96	83.9	91.8	80	78.5
MicA-ompA	96.77	100	100	100	87.5
MicA-lamB	86.36	100	90.2	76	68.4
MicC-ompC	95.24	100	69.9	52.4	80
MicF-ompF	100	96	96	86.9	80
OxyS-fhlA	83.33	89.7	66.7	54.5	66.7
RyhB-sdhD	72.73	75	74.1	79.4	82.4
RyhB-sodB	100	100	90	94.7	56.3
SgrS-ptsG	71.79	65.1	85	85	85
Spot42-galK	72	55.1	50	52.3	69
Average	89	84.5	79.1	76.3	83.6

Our genetic algorithm randomly selects the sub-diagonals to make individuals. Therefore different individuals with variety sub-diagonals are constructed. Due to the nature of proposed genetic approach, some of the RNA-RNA interactions can be predicted more accurate than the other algorithms. For example the accuracy rate of Tar-Tar* is obtained 100%, while maximum accuracy of the other approaches is 90.9%.

We compare the computational time complexity of GRNAs and state-of-the-are methods. The time and space complexity of several algorithms (TIRNA [15], App (approximation algorithm to predict SSIR) [14], ripalign [23], and other methods in the Tables 1, 2 and 3) are given in Table 4. As it is shown, the time complexity of GRNAs in each iteration is *O*(*l*^2^ + |*P*|), where *l* and |*P*| indicate sum of the length of the two RNAs and the length of an individual, respectively. Also, Space complexity of the proposed method is *O*(*l*^2^ + |*P*|).

**Table 4:** Comparison of time and space complexity of some algorithms

**Algorithm**	**Time complexity**	**Space complexity**
GRNAs	*O*(*l*^2^ + |*P*|)	*O*(*l*^2^ + |*P*|)
TIRNA	*O*(*k*^2^ log *k*^2^)	*O*(*k*^2^)
SPM	*O*(*n*^3^*m*^3^)	*O*(*n*^2^*m*^2^)
LM	*O*(*n*^3^*m*^3^)	*O*(*n*^2^*m*^2^)
inRNAs	*O*(*k*^4^*w*)	*O*(*k*^2^)
RNAup	*O*(*n*^3^*m*)	*O*(*n*^2^)
EBM	*O*(*n*^3^*m*^3^)	*O*(*n*^2^*m*^2^)
App	*O*(*n*^3^*m*^3^)	*O*(*n*^2^*m*^2^)
Pairfold	*O*(*k*^3^)	*O*(*k*^2^)
IntaRNA	*O*(*nm* + *nl*^3^)	*O*(*nm*)
ripalign	*O*(*N*^6^)	*O*(*N*^4^)
PETcofold	*O*(*MIl*^3^)	
RactIP	*O*(*n*^5^)	

Here, *l* and |P| indicate the sum of the length of two RNAs and the length of an individual, respectively.

## Conclusion

In this paper, a new genetic algorithm was introduced for solving RNA-RNA interaction prediction problem. In this algorithm, all possible stems in each RNA and hybrid regions between two RNAs are extracted from a dot matrix showing all possible base pairs. Initial population is formed based on some stems and hybrid regions of the dot matrix. Minimum free energy is considered as a fitness function. Crossover operation is done between some consecutive individuals in the population. Mutation is taken on a few randomly selected individuals. Population generation continues until the minimum free energy of the best individual becomes minimal enough. Finally, one of the best individuals is selected to form RNA-RNA interaction structure. The proposed algorithm was tested on several RNA-RNA interaction datasets. The experimental results indicate a high accuracy of GRNAs. Furthermore, time and space complexity of GRNAs is as efficient as the other related studies.

### Availability

The program of GRNAs is available at http://mostafa.ut.ac.ir/grnas.

## Competing interests

The authors declare that they have no competing interests.

## Authors’ contributions

SM participated in the design of the algorithm, performed the experiments and drafted the manuscript. FZM contributed in the design of the algorithm, performed the experiments, drafted the manuscript and supervised the project. NMC contributed in the design of the algorithm and drafted the manuscript. All authors contributed to the writing of the manuscript. All authors read and approved the final manuscript.

## References

[B1] Mneimneh S On the approximation of optimal structures for RNA-RNA interaction Trans Comput Biol Bioinform 2009 6 682 688 10.1109/TCBB.2007.70258 19875865

[B2] Alkan C Karakoc E Nadeau JH Sahinalp C Zhang K RNA-RNA interaction prediction and antisense RNA target search J Comput Biol 2006 13 267 282 10.1089/cmb.2006.13.267 16597239

[B3] Andronescu M Zhang ZC Condon A Secondary structure prediction of interacting RNA molecules J Mol Biol 2005 345 987 1001 10.1016/j.jmb.2004.10.082 15644199

[B4] Dirks R Bios J Schaeffer JM Winfree E Pierce N Thermodynamic Analysis of Interacting Nucleic Acid Strands Soc Ind Appl Math 2007 49 65 88

[B5] Rehmsmeier M Steffen P Hochsmann M Giegerich R Fast and effective prediction of microRNA/target duplexes RNA 2004 10 1507 1517 10.1261/rna.5248604 15383676 PMC1370637

[B6] Markham NR Zuker M UNAFold: Software for Nucleic Acid Folding and Hybridization Methods Mol Biol 2008 453 3 31 10.1007/978-1-60327-429-6_1 18712296

[B7] Bernhart S Tafer H Mückstein U Flamm C Peter F Stadler P Hofacker I Partition Function and Base Pairing Probabilities of RNA Heterodimers Algorithms Mol Biol 2006 1 3 10.1186/1748-7188-1-3 16722605 PMC1459172

[B8] Mückstein U Tafer H Bernhart S Hernandez-Rosales M Vogel J Stadler P Hofacker I Translational control by RNA-RNA interaction: Improved computation of RNA-RNA binding thermodynamics Bioinform Res Dev 2009 13 114 127

[B9] Mückstein U Tafer H Hackermuller J Bernhart SH Stadler PF Hofacker IL Thermodynamics of RNA-RNA binding Bioinformatics 2006 22 177 1182 10.1093/bioinformatics/btl024 16446276

[B10] Tafer H Hofacker I RNAplex: a fast tool for RNA-RNA interaction search Bioinformatics 2008 24 2657 2663 10.1093/bioinformatics/btn193 18434344

[B11] Tafer H Amman F Eggenhofer F Stadler PF Hofacker IL Fast accessibility-based prediction of RNA-RNA interactions Bioinformatics 2011 27 14 1934 1940 10.1093/bioinformatics/btr281 21593134

[B12] Kato Y Akutsu T Seki H A grammatical approach to RNA-RNA interaction prediction Pattern Recogn 2009 42 531 538 10.1016/j.patcog.2008.08.004

[B13] Salari R Backofen R Sahinalp SC Fast prediction of RNA-RNA interaction Algorithms Mol Biol 2010 5 5 10.1186/1748-7188-5-5 20047661 PMC2828455

[B14] Busch A Richter AS Backofen R IntaRNA: efficient prediction of bacterial sRNA targets incorporating target site accessibility and seed regions Bioinformatics 2008 24 2849 2856 10.1093/bioinformatics/btn544 18940824 PMC2639303

[B15] Montaseri S Moghadam-Charkari N Zare-Mirakabad F A heuristic approach to RNA-RNA interaction prediction J Theor Biol 2012 300 206 211 22300797 10.1016/j.jtbi.2012.01.025

[B16] Huang FWD Qin J Reidys CM Stadler PF Target prediction and a statistical sampling algorithm for RNA-RNA interaction Bioinformatics 2010 26 175 181 10.1093/bioinformatics/btp635 19910305 PMC2804298

[B17] Kato Y Sato K Hamada M Watanabe Y Asai K Akutsu T RactIP: fast and accurate prediction of RNA-RNA interaction using integer programming Bioinformatics 2010 26 i460 i466 10.1093/bioinformatics/btq372 20823308 PMC2935440

[B18] Seemann SE Richter AS Gesell T Backofen R Gorodkin J PETcofold: predicting conserved interactions and structures of two multiple alignments of RNA sequences Bioinformatics 2011 2 211 219 21088024 10.1093/bioinformatics/btq634 PMC3018821

[B19] Salari R Mohl M Will S Sahinalp SC Backofen R Time and space efficient RNA-RNA interaction prediction via sparse folding 2010 RECOMB'10, Research in Computational Molecular Biology

[B20] Goldberg DE Genetic Algorithms in Search, Optimization and Machine learning, Reading 1989 MA: Addison-Wesley

[B21] Holland HJ Adaptation in Natural and Artificial Systems: An Introductory Analysis with Applications to Biology 1992 MA: Control and Artificial Intelligence. MIT Press. Cambridge

[B22] Miettinen K Neittaanmaki P Periaux J Evolutionary Algorithms in Engineering and Computer Science: Recent Advances in Genetic Algorithms, Evolution Strategies Evolutionary Programming. Genetic Programming and Industrial Applications 1999 New York: Wiley

[B23] Li AX Marz M Qin J Reidys CM RNA–RNA interaction prediction based on multiple sequence alignments Bioinformatics 2011 4 456 463 21134894 10.1093/bioinformatics/btq659

